# Depressive symptoms and quality of life in patients with benign essential blepharospasm under long-term therapy with botulinum toxin

**DOI:** 10.1007/s13760-024-02658-y

**Published:** 2024-11-01

**Authors:** Bettina Wabbels, Rebecca Liebertz

**Affiliations:** Department of Ophthalmology, University Hospital of Bonn, Ernst-Abbe-Str. 2, D-53127 Bonn, Germany

**Keywords:** Blepharospasm, Botulinum toxin, Long-term, Depression, quality of life, Beck-Depression-Inventory

## Abstract

Regular and long-term injections of botulinum toxin (BoNT) are considered the first line therapy for essential blepharospasm (BEB), but no data exists on the long-term effect of this therapy on depressive symptoms and quality of life. This study aims to prospectively evaluate the long-term effects of BoNT therapy on depressive symptoms as well as on daily activities, emotional well-being and quality of life using validated questionnaires (BEB-scale, Beck`s Depression Inventory (BDI)). 86 patients diagnosed with BEB were followed up for a median of 4 years. Clinical symptoms improved significantly after BoNT-injections. Everyday activities and subjective assessment of the overall situation improved gradually under long-term BoNT therapy. Significant correlations (*p* < 0.0001; r-values between 0.498 and 0.706) were found between the BDI and items of the BEB-scale. No significant antidepressive effect of long-term BoNT therapy was found with a low median BDI total score (5/max. 63), but up to 31.3% of BEB patients had a BDI score ≥ 11, indicating clinically relevant depressive symptoms. Of these, 65.4% had no known history of depression. Although, several studies reported an antidepressant effect of botulinum toxin injections in patients with major depression, this effect does not seem to be present in patients with BEB despite clinical improvement of symptoms. A high prevalence of previously undetected depressive symptoms was found in BEB patients. As this may influence BoNT therapy success, identifying potential depressive symptoms at the time of BEB diagnosis and initiating appropriate treatment seems important.

**Supplementary Information:**

The online version contains supplementary material available at 10.1007/s13760-024-02658-y.

## Introduction

Benign essential blepharospasm (BEB) is a common form of focal cranial dystonia [1, 2]. The condition is characterized by chronic, intermittent or persistent involuntary eyelid closure due to spasmodic contractions of the orbicularis oculi muscles. Typically, the first manifestation of BEB occurs in the fifth decade of life, with women being affected twice as often as men [3]. In Europe, the prevalence of BEB is about 36 per 1, 000 000, with a high regional variance [4, 5].

The manifestations of the condition range from repeated, frequent blinking to persistent closure of both eyes. Prolonged contractions of the periorbital muscles can lead to temporary loss of vision in both eyes. This condition is tantamount to functional blindness and significantly affects the lives of patients [6].

Currently, regular and long-term injections of botulinum toxin (BoNT) are considered the first line of therapy for BEB [2, 7]. In practice, the main substances used for the treatment of essential blepharospasm in Europe and the United States are onabotulinumtoxin A (BOTOX^®^, AbbVie, North Chicago, United States of America), incobotulinumtoxin A (XEOMIN^®^, Merz GmbH & Co, Frankfurt, Germany), and abobotulinumtoxin A (Dysport^®^, Ipsen, Slough, Berks, UK) [8, 9].

Facial spasms may interfere with patients´ daily lives and interpersonal communication, are often associated with stigmatisation, and may cause psychological and social anxiety disorders, while emotional problems may in turn exacerbate facial symptoms [10–13].

In patients with major depression, several studies in recent years reported an antidepressant effect of botulinum toxin injections [14–16]. In particular, for injections in the glabella region, a mood-enhancing effect of BoNT was observed [14, 17, 18]. A previous study in Bonn found that male patients with BEB who had received additional frontalis injections of botulinum toxin (injections in the area M. procerus, M. frontalis, M. corrugator supercilii)) showed a lower Beck`s Depression Inventory (BDI) score, and thus lower depressive symptoms than male patients who had received only periorbital injections (4.5 vs. 8 points) [19].

Since BEB can be associated with considerable psychological and social burdens, which, however, are usually not yet routinely recorded during clinical diagnosis, a better understanding of the psychosocial consequences of the disease and the possible effects of BoNT therapy is essential in order to optimize both the diagnosis and the therapy accordingly in the future.

This is the first study to prospectively evaluate the short- and long-term effects of BoNT therapy on clinical symptoms, daily living skills, health related quality of life (HRQOL) as well as general satisfaction and depressive symptoms of patients with BEB.

## Materials and methods

In this study, patients with a confirmed BEB diagnosis and BoNT treatment at the University Eye Hospital Bonn were surveyed with the Blepharospasm Scale (BEB-scale; see below) and the Beck Depression Inventory (BDI) during the Botulinum Toxin Consultation in the orthoptic outpatient clinic in several interviews over a long treatment period. All consenting patients with BEB aged > 18 years with the ability to read and sufficient knowledge of German were consecutively enrolled within a one-year period; there were no exclusion criteria.

The first interview with the two questionnaires (BEB-scale and BDI) took place either before the very first BoNT injection (de novo group) or during ongoing treatment (continuous group). The second interview (using the BEB-scale and BDI) took place after at least one year of treatment in Bonn as part of their regular visits for injections. In addition, all patients were asked to complete the BEB-scale at home three weeks after the last injection (3rd interview) and to return it to the orthoptic outpatient clinic (telephone reminder in case of consent) to evaluate also short-term effects of the ongoing BoNT treatment.

Patients were briefly instructed on the questionnaires and then asked to complete them on their own. Additionally, patients were asked about acute as well as chronic psychiatric disorders and especially about pre-existing depression and any existing central nervous medication. The BEB-scale was used to collect information on clinical symptoms and associated impairments in the daily and social life over (a) at least > 1 to 5 years of treatment with botulinum toxin and (b) the immediate period of three weeks after BoNT injection, while the BDI focused on the state of mood in the longer-term course of treatment.

Data were analyzed using IBM SPSS Statistics Version 27. Due to the large number of variables, non-parametric tests were consistently used and a statement regarding statistical significance was only made in the case of highly significant p-values (*p* ≤ 0.0001). To detect correlations between questionnaire results and influencing factors, Spearmann correlation, Mann-Whitney U test, Chi-square test and Kruskal-Wallis test were used. The Wilcoxon signed-rank test was used to compare the individual surveys.

### Questionnaires: BEB-scale and BDI-scale

The BEB scale was developed by our research group to assess the severity of eyelid spasms and to specifically evaluate their impact on activities of daily living and psychosocial HRQOL as well as the general condition of the patient. According to a hemifacial spasm grading scale (HSF-7 score) designed and evaluated in our research group [20], also the BEB-scale aims to sensitively capture clinical symptoms as well as functional and psychosocial HRQOL aspects of the disease. It includes questions on the severity and frequency of clinical symptoms, their impact on activities of daily living and psychosocial HRQOL aspects, as well as an assessment of the patient’s general condition. For this purpose, the BEB-scale includes the established Jankovic Rating Scale (JRS) for assessing the severity and frequency of eyelid spasms [21]. The Global Rating Score is used to evaluate the overall situation due to the eyelid spasms; the modified Blepharospasm Disability Index (BSDI) is used to assess the impact of BEB spasms on various activities of daily living and the modified HFS-7 score evaluates their psychosocial impact [22–25]. With the exception of the JRS, all assessments are made using visual analog scales ranging from 0 (no complaints) to 100% (maximum complaints). The BEB-Scale is available for download as supplementary material. Supplementary Materials: Figure S1: Blepharospasm (BEB)-scale.

Furthermore, the globally acknowledged BDI (1978 version) was used to quantify depressive symptoms of any etiology [26, 27]. The BDI includes 21 items on depressive symptoms. For each item, between 0 and 3 points can be assigned (0 = no expression of the symptom; 3 = maximum intensity) and the individual item scores are added up to a BDI total score (max. 63 points) [28]. A BDI total score of ≥ 11 points indicates depressive symptoms [28].

Due to the sensitive information, patients were asked to answer the questionnaire for themselves and, if possible, without help from others. To quantify improvements or deteriorations between both surveys, a BDI difference was calculated from the total score of the first and the second BDI survey. A BDI difference ≥ +/- 5 points was interpreted as a clinically significant change between two surveys [29].

## Results

**Fig. 1:**
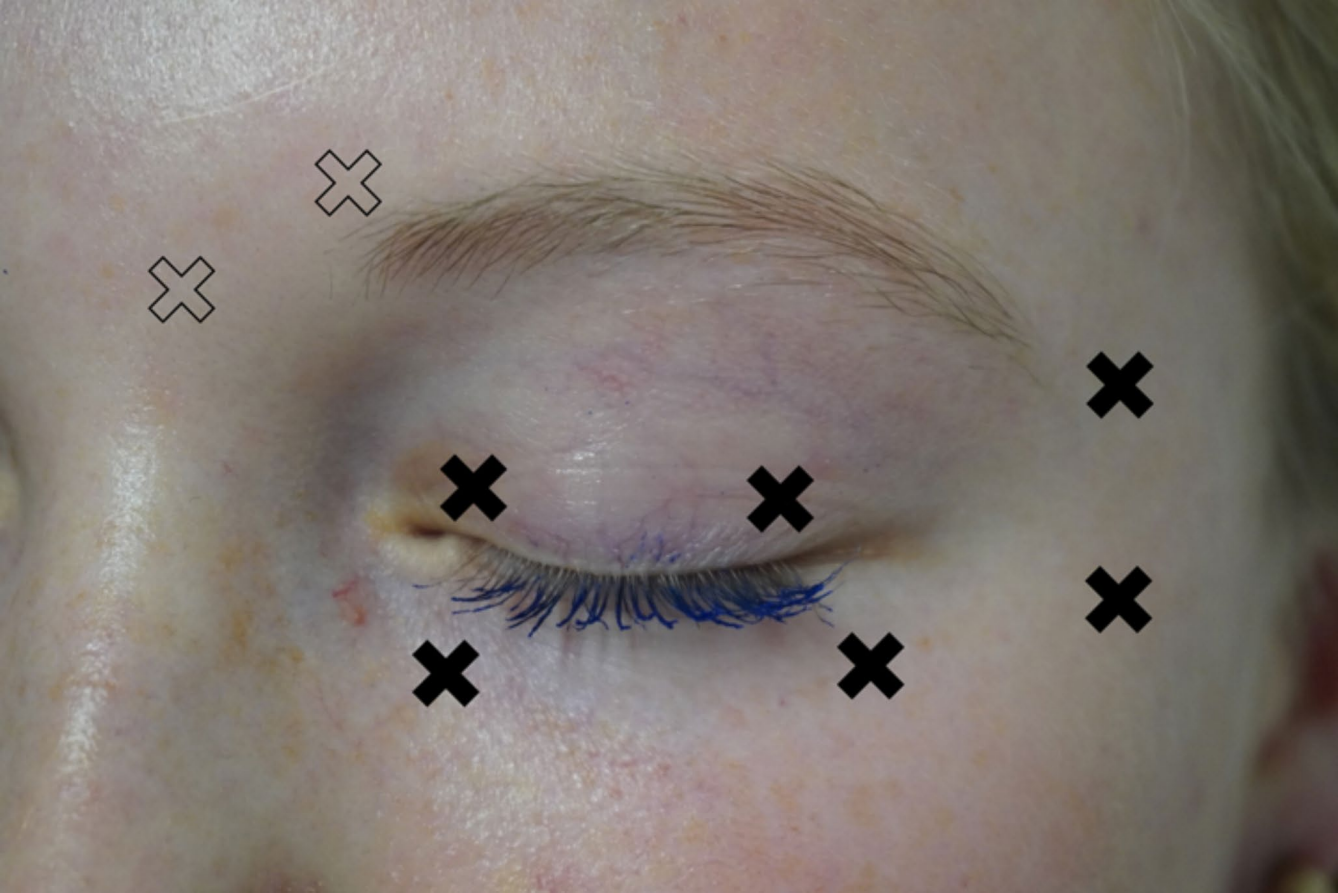
Typical periorbital BoNT-injection sites for treatment of BEB. Individual adjustment of the dosage is possible depending on patient. The black, filled crosses mark injection sites that are generally used in all patients (including the pretarsal injections on the upper eyelid), the unfilled (light) crosses in the frontalis area (Musculus corrugator and Musculus procerus) mark additional, optional injection sites. If necessary, further injection sites can be added individually, e.g. Musculus zygomaticus, Musculus nasalis, etc

A total of 86 BEB patients with a median age of 71 years (range 36–88 years) were included in the study, 29 (33,7%) were male. Thirty-seven BEB patients were assigned to the de novo group (first interview prior to beginning of BoNT therapy) and 49 to the continuous group (already under BoNT therapy at first interview, ), with both groups being comparable regarding demographic data (Table 1). The median (range) time between the first two interviews was 4 (1–5) years. For the majority of patients (*n* = 59), there was a period of 3 to 5 years between the two interviews. For 13 patients the time between the interviews was 2 years and for 14 patients less than 2 years, with a comparable distribution for both groups (Fig 1).

**Table 1:** Demographic and clinical data of both groups

	De novo group (*n* = 37)	Continous group (*n* = 49)	*p*-value
Gender, n (%)			P^1^ = 0.483
Male	14 (37.8)	15 (30.6)	
Female	23 (62.2)	34 (69.4)	
Age (years),	71 (38–88)	71 (36–85)	P^2^ = 0.879
Disease duration (years)	4 (1–18)	14 (2–38)	P^2^ < 0.0001
Treatment duration (months)	33 (12–59)	133 (12–374)	P^2^ < 0.0001
Injection cycles	11 (4–29)	43 (4-133)	P^2^ < 0.0001
Total dose (both eyes) (IE)	30 (15–80)	35 (20–80)	P^2^ < 0.0001
Frontalis injection	12 (32,4)	24 (49)	P^1^ = 0.124

n = number, p¹= significance of the chi-square test, p²= significance of the Mann-Whitney U test

A history of any psychiatric disorder, such as depression, anxiety disorders, drug dependence, schizophrenia, bipolar affective disorder or post-traumatic stress syndrome, was reported by 25.6% of all BEB patients, and depression by 18.6%. While 68% of patients with originally known depression were additionally treated continuously with antidepressants, this was not the case for patients with newly discovered depression during this study.

There was no significant difference between the de novo group and the continuous group, neither in terms of medication intake nor prevalence of depression or other previous psychiatric illnesses (chi-square test).

### BEB-scale results

#### Clinical symptoms (JRS score)

**Fig. 2:**
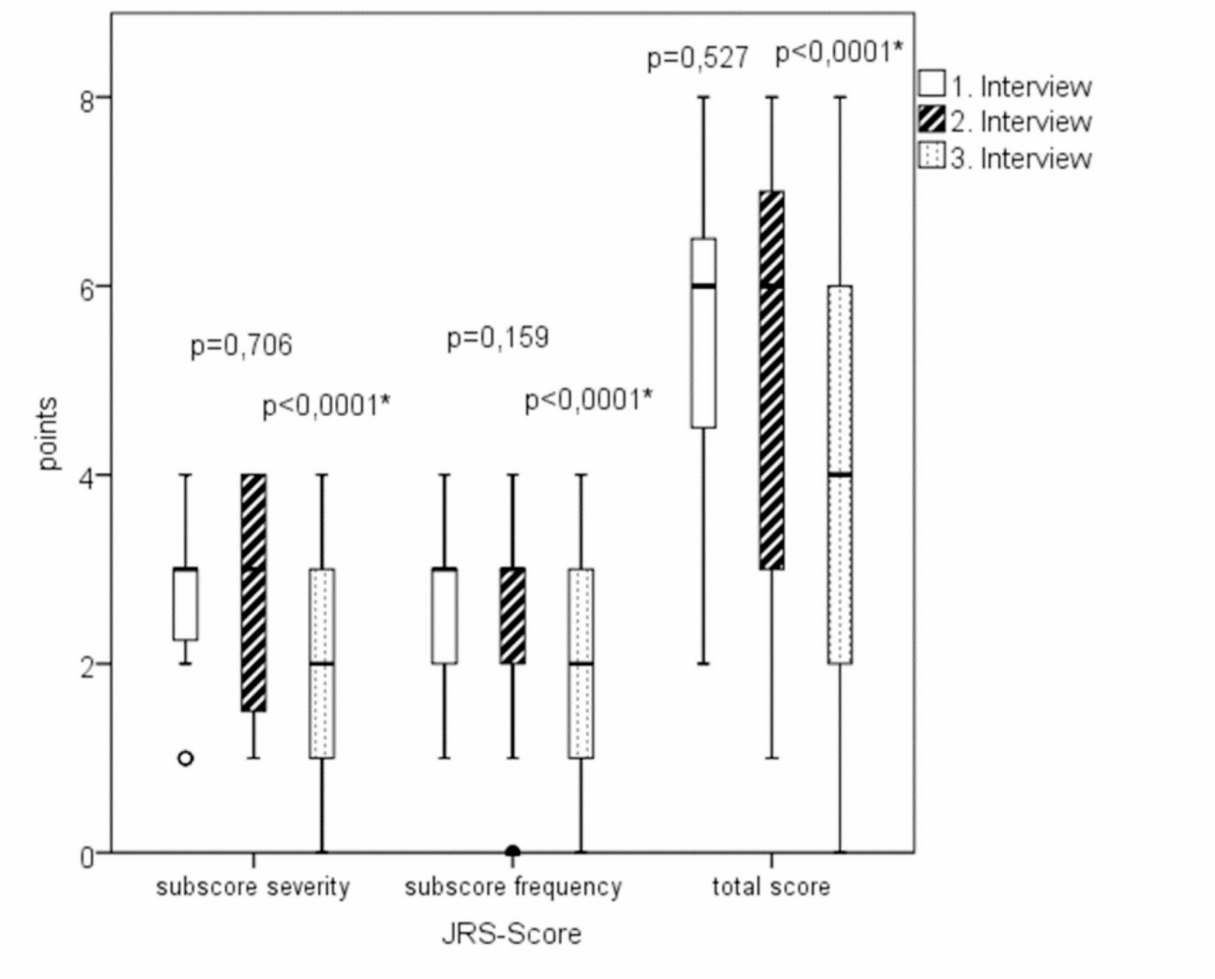
JRS scores of the three surveys in BEB patients under BoNT therapy (Wilcoxon sign rank test); boxplot with median and quartiles; °= mild outliers; maximum subscore = 4 points; maximum total score = 8 points

The JRS scores, indicating the extent of clinical symptoms, remained stable between the first and second interview and thus over a treatment period of at least one year with botulinum toxin. At the third interview, three weeks after the current BoNT injection, both the JRS total score and the JRS subscores on frequency and severity of spasms were significantly lower. This indicates the success of the BoNT therapy with clinical symptom improvement (Fig. 2).

#### Impact of eyelid spasms on functional quality of life aspects

Under BoNT therapy, the impairment of daily activities due to the eyelid spasms decreased and the overall situation improved. Over the course of the three surveys, the Global Rating Score was reduced from a median baseline value of 75–50% at the second interview and further declined to 36%at the third interview. This indicates improvement in the overall situation of the disease under BoNT therapy, which was statistically significant between the second and third interview (*p* < 0.0001; Wilcoxon). Similarly, the BSDI, measuring impairment of daily activities due to eyelid spasms, improved between first and third interview, from a mean baseline score of 50.5–36% and further to 18.8%, with a significant improvement between the second and third interview (*p* < 0.0001; Wilcoxon).

#### Impact of eyelid spasms on psychosocial quality of life aspects

**Fig. 3:**
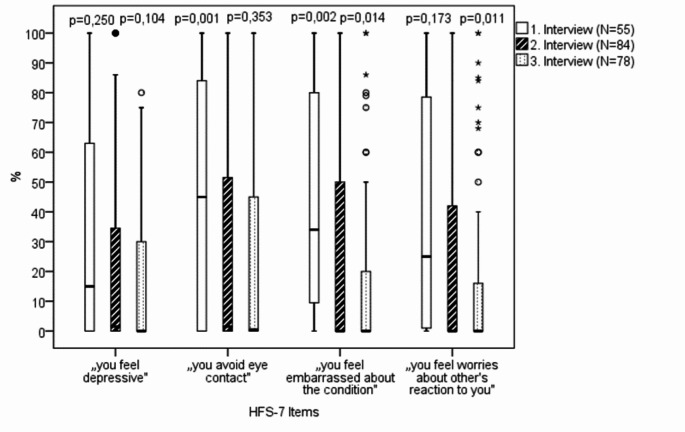
Results of the four psychosocial items of the HFS-7 at all three surveys in BEB patients (Wilcoxon signed-rank test: listwise case exclusion); boxplot with median and quartiles; N = number, p = significance, °= mild outliers, *= extreme outliers

Furthermore, there was a tendency for all psychosocial HFS-7 items queried to improve over the course of the three surveys (Fig. 3). However, due to multiple testing, this did not reach the statistical significance. At the second interview, i.e. after at least one year and in median 4 years of BoNT therapy, the median scores of all psychosocial HSF-7 items had decreased to almost 0; at the third interview three weeks after the current BoNT injection, they were zero.

No significant differences in BEB scale improvement were found between the follow-up group and de novo patients (Mann Whitney U test).

### Beck Depression Inventory

**Fig. 4:**
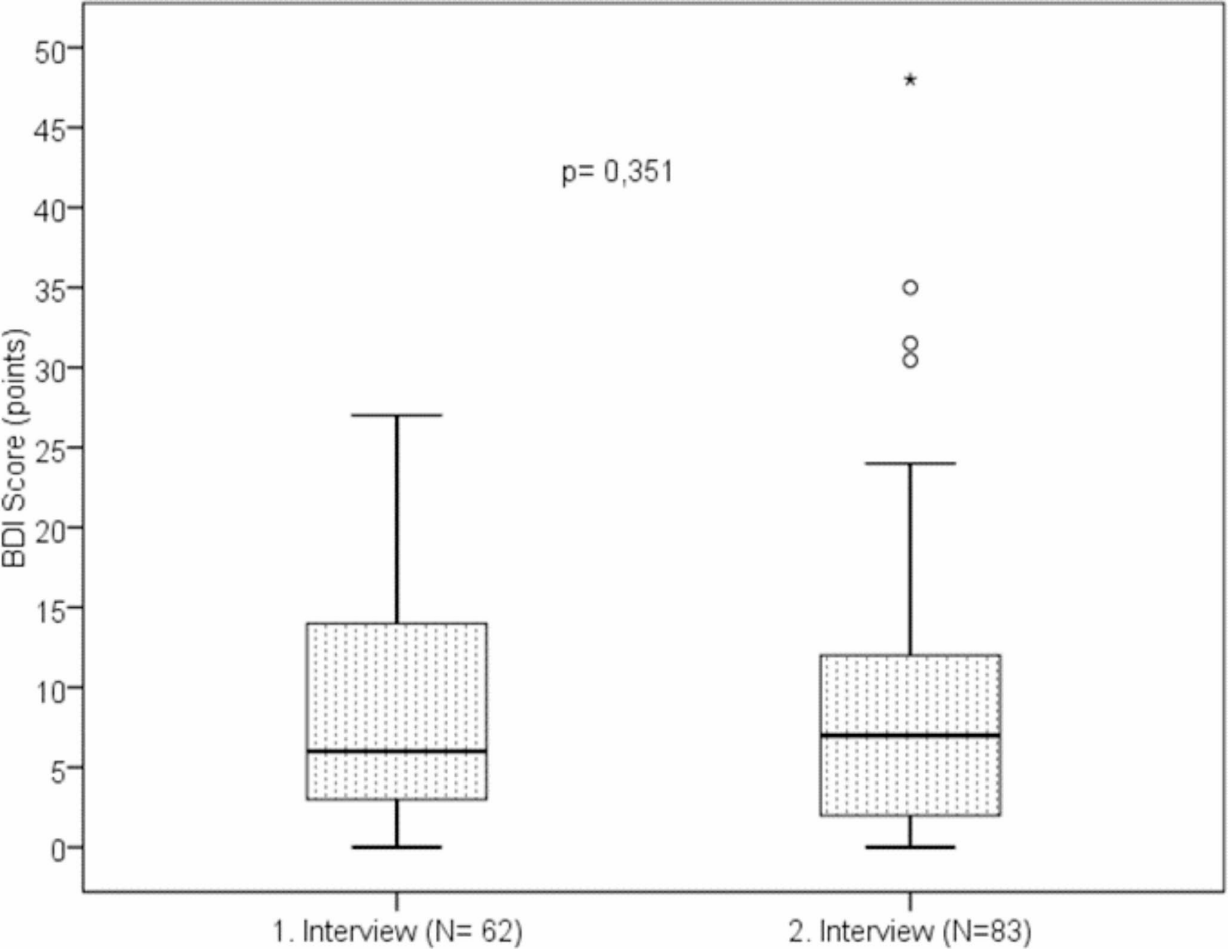
Results of the BDI in all BEB patients (Wilcoxon signed-rank test: listwise case exclusion with *N* = 59); boxplot with median and quartiles; p = significance, N = number, °= mild outliers; *= extreme outliers; maximum score BDI = 63 points

Depressive symptoms of any aetiology were assessed at the first interview and after at least one year of BoNT therapy in Bonn (second interview) with the Beck Depression Inventory (BDI). The results for the total cohort are shown in Fig. 4.

The median BDI of the total cohort at the first interview was 5 points and hence considerably lower than a score of 11 points, which is considered indicative of depressive symptoms [28]. Moreover, the BDI score did not change significantly between the first and the second interview. This indicates overall only minor depressive symptoms and a stable situation over at least one year (in median 4 years) of BoNT therapy in this cohort of BEB patients.

The individual analysis of all clinically significant changes (individual BDI difference between both questionnaires) showed no clinically relevant change in BDI in 46.5% of patients. A clinically significant improvement (difference ≥ 5 points) was observed in 11.6% of patients, and a clinically significant deterioration in 10.5% (difference ≤ -5 points). For 27 patients (31.4%), one of the two questionnaires was missing and no BDI difference could be calculated.

Twenty-six (31,3%) BEB patients had a BDI score ≥ 11 points at the second interview, indicating depressive symptoms [28]. However, 17 of these 26 patients (34.6%) with a BDI score ≥ 11 points had no known history of depression. No significant differences were found between the follow-up group and de novo patients (Mann Whitney U test).

### Correlation of BEB-scale and BDI

Overall, there was a high positive correlation between the results of the BDI and the results of a patient’s BEB-scale in both surveys. Especially at the second interview, significant correlations (*p* < 0.0001; r-values ranging from 0.498 to 0.706) were found between the BDI and the different subscales of the BEB-scale (i.e. JRS, Global Rating Score, total BSDI, HFS-7 items) (Table 2). This indicates a high association between depressive symptoms and more severe clinical symptoms or a stronger impairment of everyday functions due to BEB.

**Table 2:** Spearman correlations between the BDI score and items of the BEB-scale in patients with BEB; r = correlation coefficient; *significant correlations (*p* < 0.0001)

	BDI-Score	
	Interview 1 (baseline)	Interview 2 (median after 4 years)
**JRS-Score**	*r* = 0.352	*r* = 0.545
	*p* = 0.072	***p*** < 0.0001*
**Global Rating**	*r* = 0.541	*r* = 0.579
	*p* = 0.002	***p*** < 0.0001*
**BSDI**,** total score**	*r* = 0.593	*r* = 0.642
	***p*** < 0.0001*	***p*** < 0.0001*
**HSF-7 Items**		
You feel depressive	*r* = 0.775	*r* = 0.706
	***p < 0,*** 0001*	***p*** < 0.0001*
You avoid eye contact	*r* = 0.546	*r* = 0.498
	*p* = 0.001	p < 0.0001*
You feel embarrassed about the condition	*r* = 0.677	*r* = 0.575
	p < 0.0001*	p < 0.0001*
You feel worries about other`s reaction to you	*r* = 0.498	*r* = 0.584
	*p* = 0.003	p < 0.0001*

## Discussion

This is the first study that prospectively evaluated short- and long-term effects of BoNT therapy on clinical symptoms, HRQOL and depressive symptoms in patients with BEB. The study confirms significant improvement in clinical symptoms, everyday functioning and the subjective assessment of the overall situation of patients with BEB receiving BoNT therapy. In addition, an association was found between the severity of spasms and its resulting impairments in daily life and the level of psychosocial burden in BEB patients. At the same time, any existing depressive symptoms did not change significantly over at least one year (in median 4 years) of BoNT therapy. Accordingly, it can be assumed that the mental and emotional status of BEB patients remains stable over a longer period of BoNT therapy. However, only mild depressive symptoms were present in the majority of patients in this cohort, so there was not much potential for improvement following BoNT therapy.

It has to be emphasized, however, that the rate of existing depression among BEB patients was twice that of the general population [30]. In addition, about one third of the BEB patients showed evidence of depressive symptoms, even though the majority of these patients had no previous history of known depression. Moreover, the severity of depressive symptoms in BEB patients, assessed by BDI, was associated with a reporting of more severe and more pronounced functional impairment due to spasms. Also, patients with depressive symptoms rated their overall situation more negatively.

### Impact on clinical symptoms and quality of life

In the current study, a significant reduction of the JRS total score including both its subscores (severity & frequency) was achieved three weeks after a BoNT injection with a low median total dose of 30 IU for both eyes. Furthermore, gradual improvements were reported for the overall situation as well as in functioning of daily activities. Also, psychosocial aspects tended to improve under BoNT therapy (Fig. 2). Comparable results regarding the JRS score were consistently reported in other studies [9, 31, 32]. In addition, in agreement with our results, Kollewe et al. also observed, stable clinical symptoms over years in the long-term course under BoNT therapy [9, 33].

### Impact on depressive symptoms

No significant antidepressant effect (based on BDI score) was detected in BEB patients over a longer BoNT treatment period (minimum 1 year, median 4 years). However, it has to be noted that the low BDI score at baseline only allowed a small potential for improvement and that comparative data regarding BDI is missing for 27 patients in this study.

Although there is some evidence in the literature of a mood-enhancing effect -in addition to paralytic effects- of botulinum toxin, it has to be emphasized that the subjects in these studies suffered from clinically manifest depression [14, 16–18]. In contrast, patients in the current study received BoNT therapy for the treatment of BEB, and only mild to at best moderate depressive symptoms were present in the majority of patients, which limits the comparability of the studies.

The effect of BoNT therapy on HRQOL and/or depressive symptoms was also investigated in patients with BEB (Table 3) [10, 12, 34–43]. However, these were either cross-sectional studies with only a single survey after some years of BoNT therapy or short-term studies with a maximum follow-up of 3–4 months. In contrast, the present study prospectively collected long-term data on the basis of two surveys using the BDI depression scale, with a median time span of 4 years between the two surveys.

**Table 3:** Overview of studies on depressive symptoms in patients with BEB undergoing BoNT treatment

References	Study design	Patients with BEB	Questionnaires used	Time of the interview during BoNT therapy	Outcome /Conclusion
Present study	prospective	86	BDI BEB-scale [including Jankovic Rating Scale (JRS), modified Blepharospasm Disability Index (BSDI) psychosocial HFS-7 items]	Twice within a median of 4 years twice within a median of four years and another three weeks after current BoNT injection	No significant antidepressive effect of long-term BoNT therapy 31.3% of BEB patients with a BDI score ≥ 11 points, and thus clinically relevant depressive symptoms clinical symptoms remained stable over four years of treatment and improved significantly three weeks after current injection; overall situation and quality of life improved gradually
Hirunwiwatkul et al. 2023	prospective	184	EQ-5D-5 L (thai version) NEI-VFQ-25 JRS	Thrice: Baseline, 4–6 weeks and 12–16 weeks after BoNT	Quality of life (EQ-5D-5 L and NEI-VFQ-25) improved significantly 1 and 3 months after injection Clinical symptoms (JRS) improved significantly after one month and increased slightly again at three months after injection
Tang et al. 2019	Prospective	87	WHOQOL-BREF (chinese version) OSDI RSES	Twice: baseline and four weeks after BoNT	The domains overall QOL score, general health status, physical and psychological of the WHOQOL-BREF scale improved significantly four weeks after BoNT Clinical symptoms (OSDI) and self-esteem (RSES) also improved significantly after four weeks
Dong et al. 2018	Prospective	90	SAS SDS	Twice: directly before injection and after two months	Depressive symptoms (SDS) and anxiety (SAS) were reduced significantly 2 months after BoNT
Weiss et al. 2018	Prospective	55	EQ-5D-5 L questionnaire on life satisfaction BDI subjective rating scale of severity pain scale PSQI	Twice: three months after regular injection and four weeks after reinjection	Significant improvement in clinical symptoms, but no improvement in quality of life, sleep quality, pain, depressive symptoms and life satisfation
Ochudlo et al. 2007	Prospective	33	SF-36 MADRS UDRS	Twice: baseline and one month after BoNT	Quality of life (SF-36) improved about 43,76% after injection Reduction of depressive symptoms (MADRS) about 55,68% after injection Clinical Symptoms (UDRS) also improved after one month
Müller et al. 2002	Prospective	89	SF-36 BDI Global impression scale	Twice: directly before injection and after four weeks	Directly before injection BEB patients had lower life quality compared to normal population; 37% of BEB patients were depressive at baseline (BDI ≥ 10); Reduction of global impression scale of 42,2% after four weeks. No improvement in quality of life (SF-36) or BDI after BoNT
Kako et al. 2021	Cross-sectional study	27	Effectiveness score home QOL social QOL HADS (japanese version)	Once after several injections (minimum 3) of botulinumtoxin; no details regarding exact timing	Definite depression in HADS is significantly more common in patients with blepharospasm than in patients with hemifacial spasm quality of life at home (home QOL) and at social settings (social QOL) in BEB patients were lower compared to patients with hemifacial spasm
Lawes-Wickwar et al. 2021	Cross-sectional study	76	JRS BSDI HADS DAS24 IPQ-R TRI CDQ-24	Once after several injections (minimum 2) of treatment, interview before reinjection	The disease had a huge impact on activities of daily living and perceived stigma Overall quality of life (CDQ-24) correlated significantly with lower injection dose, less appearance-related distress, less negative emotional beliefs, better mood and less negative consequences of having the condition
Streitová et al. 2014	Cross-sectional study	9	JRS patient´s questionnaire with 11 items	Once after a treatment with BoNT for 15–20 years (mean 18,2)	Quality of life improved by 71%; most impact on the aspect social communication
Setthawatcharawanich et al. 2011	Cross-sectional study	32	Thai HFS-30 Thai Depression Inventory	Once: patients who are under treatment at least 4,5 years (exact timing of interview not detailed)	Quality of life (HFS-30) was lower in BEB patients than in the healthy controll group or patients with hemifacial spasm HFS-30 correlated positively with depressive symptoms
MacAndie et al. 2004	Cross-sectional study	44	GBI	Once after BoNT injection, sent by post to be completed within 4 weeks	81,8% of patients reported benefit of BoNT and a significant improvement in quality of life
Tucha et al. 2001	Cross-sectional study	51	Quality of life scale focusing on emotional and social well-being	Once after several years of treatment (1–7 years, mean 33,4 months)	Nearly all patients reported benefit of BoNT referring quality of life;

BDI, Beck`s Depression Inventory; BEB, benign essential blepharospasm, WHOQOL-BREF, short version of WHOQOL-100 questionnair; OSDI, Ocular Surface Disease Index; RSES, Rosenberg-Selfesteem-Scale; EQ-5D-5 L, European Quality of Life Five Dimension Five levels ; NEI-VFQ-25, 25-item National Eye Institute visual function questionnaire (thai version) ; JRS, Jankovic rating scale; EQ, EuroQol; PSQI, Pittsburgh sleep quality index; BSDI, Blepharospasm Disability Index; HADS, Hospital anxiety and depression scale; DAS24, Derriford Appearance Scale; IPQ-R, revised illness perceptions questionnaire; TRI, Treatment representations inventory; CDQ-24, Craniocervical dystonia questionnaire; GBI, Glasgow benefit inventory; SF-36, Short Form-36 Health Survey; MADRS, Montgomery–Åsberg Depression Rating Scale; UDRS, Unified Dystonia Rating Scale; HFS-30, health-related quality of life questionnaire for patients with hemifacial spasm ; SAS, self rating anxiety scale; SDS, self rating depression scale

Consistent with previous research, our study also demonstrates that clinical symptoms improve with BoNT therapy. However, the effects of BoNT therapy on HRQoL vary depending on the questionnaire and study design [10, 12, 34–43]. Depressive symptoms are reported more frequently in BEB than in the general population. However, this is the first study to demonstrate that these symptoms may persist for years despite successful treatment.

In our patient cohort, the total score of the BDI at baseline was low with a median of about 5 (out of a maximum of 63) [26, 27] and data for comparing BDI scores were missing for 27 patients in our study. Therefore, due to in median the hardly present or relatively low depressive symptoms, there was not much potential for improvement by BoNT therapy. This is also supported by the results of the psychosocial items of the HSF-7, which were already close to 0 at the second survey and zero at the third survey, so that further improvements could not be measured.

Consistent with the study by Müller et al., who reported a BDI ≥ 10 points in 37% of patients, our research also found that women reported higher overall BDI scores than male participants, suggesting that women are more likely to experience the symptoms and consequences of BEB in their daily lives [12].

### Correlation of clinical and depressive symptoms

The depressive symptoms in the overall cohort of BEB patients in this study assessed with the BDI correlated significantly positively with the severity of the spasms (JRS scores) and the patients’ subjective assessment of their overall situation (Global Rating Score). An association between emotional well-being and clinical manifestation of BEB can therefore be assumed. Furthermore, the current study shows a positive correlation between depressive symptoms (BDI) and impairments due to spasms in daily life (total BSDI) of BEB patients. A study by Junker et al. on 603 patients with isolated dystonia (including 41 patients with BEB) showed that depressive symptoms and anxiety were associated with a reduced quality of life and severe dystonia symptoms were associated with poorer physical and social functioning [44]. An association with depressive symptoms has also been described for dyskinesias, such as Parkinson’s disease, and for visual impairment [45−48].

The extent of the patients’ psychosocial stress, as measured by the individual HFS-7 items, was also associated with stronger depressive symptoms assessed in the BDI. It seems plausible that the facial spasms of BEB can affect interpersonal communication and may lead to decreased self-confidence, stigmatization, social disadvantage, and in the course also eventually reactively to depressive symptoms. However, the BDI scale is not an appropriate tool to distinguish primary from reactive depression [28]. In addition, the onset of depressive symptoms was not recorded in this study, and therefore no conclusion can be drawn about the extent to which depressive symptoms and spasms correlate in time. Consequently, no statement can be made as to whether the presence of a BEB led to depressive symptoms or whether, due to a pre-existing depressive mood, the clinical, functional, and psychosocial consequences of a BEB may be perceived as more limiting.

### BEB Patients with depression

In the current study, 18.6% of the BEB patients had a history of additional depression. Thus, the prevalence of depression among the BEB patients in this study is almost twice as high as in the general population in Germany (9.3%), while the prevalence of psychiatric disorders was comparable to that in the general population (24.3% vs. 27.7%) [30]. This is in line with other studies that also describe a higher prevalence of depression in patients with BEB than in the general population [12, 49, 50]. In addition, in our study, a considerable proportion of the BEB patients (31.3%) had a BDI score ≥ 11 points, and thus clinically relevant depressive symptoms. The majority of these patients (65.4%) had no previous history of depression. Accordingly, as in the general population [50], there was a high proportion of undiagnosed and thus untreated depression within the BEB patients in the current study, with depressive symptoms remaining unchanged over a median of 4 years.

### Limitations

Due to the explorative design of the study, a high number of statistical tests were required, which in turn led to an overall low level of significance due to multiple testing. Some patients (27%) also required assistance in completing the questionnaires due to visual impairments or age-related deficits. In these cases, the items were read aloud during the interview so that the patient could make his or her selection. Although these personal interviews took place in a separate room, an influence on the results due to the missing self-editing cannot be excluded.

### Future implications for clinical practice and research

Overall, our data suggest that accompanying depressive symptoms are of great importance in BEB patients and should not be underestimated, as they can impair the quality of life and possibly also the perception of the BoNT therapy success. In view of the high prevalence of unrecognized depressive symptoms in BEB patients, it therefore seems essential to communicate this topic with patients. In addition, standardized screening for possible depression should also be carried out at the time of diagnosis of BEB and, if necessary, appropriate treatment should be initiated. According to the results of our study, BoNT therapy alone is not expected to improve depressive symptoms.

In terms of future research, it seems important to investigate which depression scale is best suited to assess symptoms of depression and anxiety in patients with BEB. For example, the HADS-D, which comprises 14 items and was developed as an instrument for detecting anxiety and depression in individuals with any physical illnesses, may prove to be a better screening tool [51]. Another important field of research might be to investigate whether antidepressant therapy could potentially support and complement the BoNT treatment of BEB.

## Conclusions

Overall, our study including 86 patients with BEB confirmed that clinical symptoms significantly improved following BoNT injections. A long-term BoNT therapy of in median 4 years gradually reduced impairment of daily activities and improved the overall situation due to eyelid spasms, but did not show a significant anti-depressive effect. There was a strong positive correlation between depressive symptoms (assessed by BDI) and severity of clinical symptoms (measured by JRS) and their impact on daily activities, overall situation and psychosocial measured by (Global Rating Score, total BSDI, HFS-7 items).

A high prevalence of previously undetected depressive symptoms (31.3%) was found in BEB patients. Patients with depressive symptoms experienced more severe clinical symptoms and functional limitations due to spasms. As this may affect the success of BoNT therapy, it seems important to identify possible depressive symptoms at the time of BEB diagnosis and to initiate appropriate treatment.

## Electronic supplementary material

Below is the link to the electronic supplementary material.

Supplementary Material 1

## Data Availability

No datasets were generated or analysed during the current study.
