# Supplementary material for: Depressive symptoms and quality of life in patients with benign essential blepharospasm under long-term therapy with botulinum toxin
Source: Acta Neurol Belg. 2024 Nov 1;125(1):157–68. doi: 10.1007/s13760-024-02658-y (PMC11876193; doi:10.1007/s13760-024-02658-y)
Supplement: Supplementary file 1 — Supplementary Material 1 [file 13760_2024_2658_MOESM1_ESM.pdf]

Supplemental Material

Acta Neurologica Belgica

Depressive symptoms and quality of life in patients with benign essential blepharospasm under long-term therapy with botulinum toxin

Bettina Wabbels[1], Rebecca Liebertz[1,2]

1 Department of Ophthalmology, University Hospital of Bonn, ORCID 0000-0002-7767-114X

2 Department of Neurology, LVR Clinic Bonn, ORCID 0009-0001-1596-5513

Corresponding author:

Bettina Wabbels

Department of Ophthalmology,

University Hospital of Bonn,

Ernst-Abbe-Str. 2

D-53127 Bonn, Germany;

E-Mail: bettina.wabbels@ukbonn.de

Blepharospasm-Scale

1. Jankovic-Score

Severity	Frequency
0 = None<br>1 = Minimal, increased blinking present only with external stimuli (e.g., bright light, wind, reading, driving, etc.)<br>2 = Mild, but spontaneous eyelid fluttering (without actual spasm), definitely noticeable, possibly embarrassing, but not functionally disabling)<br>3 = Moderate, very noticeable spasm of eyelids only, mildly incapacitating<br>4 = Severe, incapacitating spasm of eyelids and possibly other facial muscles	0 = None<br>1 = Slightly increased frequency of blinking<br>2 = Eyelid fluttering lasting less than 1 second in duration<br>3 = Eyelid spasm lasting more than 1 second, but eyes open more than 50% of the waking time<br>4 = Functionally "blind" due to persistent eye closure (blepharospasm) more than 50% of the waking time

Score: ____

2. Global rating

Free of complaints suffering extremely

0% I____I 100%

3. Blepharospasm Disability Index (BSDI)

(1) **Reading:** O not applicable

No limitation not possible due to BEB

0% I____I 100%

(2) **Driving:** O not applicable

No limitation not possible due to BEB

0% I____I 100%

(3) **Watching television / movie:** O not applicable

No limitation not possible due to BEB

0% I____I 100%

(4) **Going shopping:** O not applicable

No limitation not possible due to BEB

0% I____I 100%

(5) **Daily activities:**

No limitation not possible due to BEB

0% I____I 100%

(6) **Taking a walk:** O not applicable

No limitation not possible due to BEB

0% I____I 100%

4. HFS-7 Items

(1) Felt depressed

0% I____I 100%

(2) Avoided eye contact

0% I____I 100%

(3) Felt embarrassed about having the condition

0% I____I 100%

(4) Felt worried about others' reactions to you

0% I____I 100%
